# The use of poly(N-[2-hydroxypropyl]-methacrylamide) hydrogel to repair a T10 spinal cord hemisection in rat: a behavioural, electrophysiological and anatomical examination

**DOI:** 10.1042/AN20120082

**Published:** 2013-05-30

**Authors:** Vincent Pertici, Julien Amendola, Jérôme Laurin, Didier Gigmes, Laura Madaschi, Stephana Carelli, Tanguy Marqueste, Alfredo Gorio, Patrick Decherchi

**Affiliations:** *Aix-Marseille Université (AMU), UMR CNRS 7287, ‘Institut des Sciences du Mouvement (ISM)–Etienne-Jules MAREY’ Equipe ‘Plasticité des Systèmes Nerveux et Musculaire’ Parc Scientifique et Technologique de Luminy, Faculté des Sciences du Sport de Marseille CC910-163 Avenue de Luminy F-13288, Marseille Cedex 09, France; †Aix-Marseille Université (AMU), UMR CNRS 7273, ‘Institut de Chimie Radicalaire’ (ICR) Equipe ‘Chimie Radicalaire Organique et Polymères de Spécialité’, Case 562-Avenue Escadrille Normandie-Niemen F-13397, Marseille Cedex 20, France; ‡Dipartimento di Medicina, Chirurgia e Odontoiatria Faculta di Medicina e Chirugia, Università degli Studi di Milano, Ospedale San Paolo, Via Antonio di Rudinì, 8 20142 Milano, Italy

**Keywords:** biomaterial, ED1, monosynaptic Ia afferent reflex, motoneuron recruitment, myelin, neurofilament, pHPMA, spasticity, CNS, central nervous system, DAPI, 4′,6-diamidino-2-phenylindole, EIF, electrically induced muscle fatigue, EMG, electromyographic, GABA, γ-amino-butyric acid, PBS, phosphate buffer saline, pHPMA, poly N-(2-hydroxypropyl)-methacrylamide, pHEMA, poly(2-hydroxyethyl methacrylate), PGA, poly(glycolic), PLA, poly(lactic acid), SCI, spinal cord injury

## Abstract

There have been considerable interests in attempting to reverse the deficit because of an SCI (spinal cord injury) by restoring neural pathways through the lesion and by rebuilding the tissue network. In order to provide an appropriate micro-environment for regrowing axotomized neurons and proliferating and migrating cells, we have implanted a small block of pHPMA [poly N-(2-hydroxypropyl)-methacrylamide] hydrogel into the hemisected T10 rat spinal cord. Locomotor activity was evaluated once a week during 14 weeks with the BBB rating scale in an open field. At the 14th week after SCI, the reflexivity of the sub-lesional region was measured. We also monitored the ventilatory frequency during an electrically induced muscle fatigue known to elicit the muscle metaboreflex and increase the respiratory rate. Spinal cords were then collected, fixed and stained with anti-ED-1 and anti-NF-H antibodies and FluoroMyelin. We show in this study that hydrogel-implanted animals exhibit: (i) an improved locomotor BBB score, (ii) an improved breathing adjustment to electrically evoked isometric contractions and (iii) an H-reflex recovery close to control animals. Qualitative histological results put in evidence higher accumulation of ED-1 positive cells (macrophages/monocytes) at the lesion border, a large number of NF-H positive axons penetrating the applied matrix, and myelin preservation both rostrally and caudally to the lesion. Our data confirm that pHPMA hydrogel is a potent biomaterial that can be used for improving neuromuscular adaptive mechanisms and H-reflex responses after SCI.

## INTRODUCTION

An SCI (spinal cord injury) induces axonal degeneration followed by cellular reactions that lead to a glial scar formation that forms a tight barrier and to post-traumatic pseudocyst cavities. All of these reactions induce network disorganization and mechanically hinder the axonal regeneration of injured CNS (central nervous system) neurons (Berry et al., 1983; Fitch et al., 1999). Furthermore, the glial response involves microglia, meningeal cells, astrocytes and oligodendrocyte precursors, the majority of which produce molecules known to inhibit the axonal elongation (Fawcett and Asher, 1999).

One of the methods to limit the glial scar formation is to re-form the tissue structure and to promote axonal regeneration across such CNS by the use of biomaterial-based scaffolds to bridge the lesion site. Scaffolds may serve as a matrix for cell growth within the inhibitory region of the injury, may allow the diffusion of various neuroactive substances including growth factors and may provide a permissive environment supporting the regrowth of axons through the lesion site.

The use of various biomaterials has been proven to be effective in providing a cellular framework for regenerating tissue (Kliot et al., 1990; Woerly, 1993; Woerly et al., 1993; Xu et al., 1995; Meek et al., 2001; Oudega et al., 2001; Wan et al., 2001; Ahmed et al., 2004; Cai et al., 2005; Stang et al., 2005). Most of these biomaterials used in tissue engineering studies published to date have been based on PGA [poly(glycolic)], PLA [poly(lactic acid)] and their copolymers. Although they have a wide acceptance, these polymers exhibit some disadvantages: (i) they are not elastomeric, (ii) they are hydrophobic, (iii) they break down into acidic products that are incompatible with cell growth, (iv) the acidic breakdown products autocatalyse further polymer breakdown, often leading to catastrophic disintegration of larger masses of polymer, and (v) they are difficult to derivate chemically.

Non-resorbable polymers are suitable implantation materials. Acrylate and methacrylate polymers are tolerated *in vivo*. These polymers with hydrophilic substitute groups can form hydrogels that are suitable in medicine and biology applications because of their high water content and favourable mechanical properties. Indeed, they are water-swollen polymers with a macromolecular three-dimensional network that resembles a sponge. Moreover, they are also stable, biocompatible, with a viscoelasticity close to living tissues, with a porosity allowing neuroactive molecules, nutriments and metabolites to permeate through the matrix, and have the ability to entrap cells that act as a source of extracellular matrix (Atzet et al., 2008). Among candidates, hydrogels of pHEMA [poly(2-hydroxyethyl methacrylate)] are known to present the previously cited characteristics. They can be fabricated in various architectures with mechanical properties that are similar to natural tissue (Homsy, 1970; Ratner and Miller, 1973; Peppas et al., 1985; Metters et al., 1999; Pradny et al., 2002; Bakshi et al., 2004; Pradny et al., 2005; Atzet et al., 2008). Thus, these hydrogels are attractive materials for axonal regeneration in the CNS because macroporous pHEMA hydogels can be synthesized using a variety of chemistries, resulting in a range of microstructures with varying degrees of porosity and pore size in which injured axons should regrow, i.e. they can be used as bridging structures providing physical as well as trophic support for the regenerative process. Hydrogels based on crosslinked pHPMA [poly N-(2-hydroxypropyl)-methacrylamide] are also known to have the potential for facilitating tissue regeneration in the injured spinal cord (Woerly, 2000; Woerly et al., 2001a, 2001b, 2001c, 2004, 2005; Sykova and Jendelova, 2007), the brain (Lesny et al., 2002) and the optic tract (Loh et al., 2001). This pHPMA provides a porous environment into which cells from the intact neural parenchyma can migrate.

It is well established that, after a trauma, many differentiated neurons trans-synaptically degenerate below and above the lesion, but some motoneurons survive and remain functional (Kaelan et al., 1988; Eidelberg et al., 1989; Bjugn et al., 1997; Chang, 1998; Jimenez et al., 2000; Button et al., 2008). As a result, spinal segments that are not fully injured remain able to exhibit functional activity mediated by reflex pathways. The Hoffman (H)-reflex, which is the electrical analogue of the monosynaptic stretch reflex, can be used to assess the excitability of motoneuron pools (Hunt, 1955; Gozariu et al., 1998; Manjarrez et al., 2000; Chen et al., 2002). More precisely, it provides information on the functional properties of the Ia afferents and homonymous α-motoneurons, under physiological and pathological conditions (Pierrot-Deseilligny and Mazevet, 2000). It is a commonly used clinical tool (Misiaszek, 2003; Yablon and Stokic, 2004) that yields information about altered properties of the reflex after SCI. The H-reflex is elicited by an electrical stimulation of a peripheral nerve that innervates the muscle from which the reflex is recorded. A single stimulus generates a short-latency M-wave, resulting from direct stimulation of the motor axons, and a long-latency H-wave elicited by the activation of α-motoneurons activated by Ia afferents (Gozariu et al., 1998). The amplitude of the H-reflex has been shown to be proportional to the number of activated motoneurons (Magladery and Teasdall, 1958) and the reflex represents a good measure of the transmission between muscle afferents and motoneurons (Magladery et al., 1951a, 1951b; Burke et al., 1984). In normal subjects, the H-wave amplitude remains constant (Hasegawa and Ono, 1996a, 1996b) and a depression is observed when frequency stimulation increases. However, after SCI, H-reflex increases in amplitude (Malmsten, 1983) and the decrease in H-wave amplitude becomes less sensitive to stimulus frequency, resulting in a higher H/M ratio compared with corresponding frequencies in uninjured control (Thompson et al., 1992; Calancie et al., 1993). Such phenomenon results in a decrease of the presynaptic inhibition by local interneurons on Ia afferents following loss of descending projections from the brainstem to those interneurons (Delwaide et al., 1973). The elevated H-reflex amplitude and H/M ratio at the baseline stimulus frequency are manifestations of hyperexcitability (Delwaide et al., 1973) and spasticity (Diamantopoulos and Zander Olsen, 1967; Olsen and Diamantopoulos, 1967; Taylor et al., 1984) and are used to correlate reflex pathway properties with functional outcome in animal experiments (Lee et al., 2005).

In the present study, outcomes of pHPMA hydrogel were also evaluated during muscle exhaustive exercise. Adjustment to exercise is a complex phenomenon that could be briefly described as an increase in oxygen and energetic metabolites supply to working muscle. It is commonly admitted that exercise induces an increase in HR (heart rate), MABP (mean arterial blood pressure) and minute ventilation by two classes of neural mechanisms: (i) the central command system which involves the direct activation of brainstem locomotion, ventilatory and autonomic networks by the cerebral processes occurring at the onset of exercise (Krogh and Lindhard, 1913; Goodwin et al., 1972; Eldridge et al., 1985) and (ii) the reflex network resulting of the activation of a complex system of feedback and feed forward through medullar pathways (Iwamoto et al., 1985; Rowell and O’Leary, 1990; Kaufman and Hayes, 2002). Recently, we demonstrated that this reflex is mediated by metabosensitive afferent fibres from groups III and IV whose receptors are uncapsulated nerve endings found in the connective tissue and in the walls of small vessels within the muscle respectively (Decherchi et al., 2007). We also showed that metabolites released from a low-frequency (10 Hz) electrically stimulated muscle-activated nerve endings that elicited a reflex increase in blood pressure, sympathetic activity and ventilation, in animals whose humoral communication was abolished. These reflexes are named ‘Exercise or Muscle Reflex’ (ER or MR) or “Metaboreflex”. Afferent inputs from exercising skeletal muscle are believed to signal the CNS that blood and/or oxygen supply to the metabolically active tissues is not adequate to meet blood and/or oxygen demand (Mitchell, 1985; Rowell and O’Leary, 1990). Consequently, metabolic stimuli provide an error signal indicating that blood supply and demand in contracting muscle are not matched properly. After a spinal cord trauma, the metaboreflex originating from skeletal muscle and contributing significantly to the regulation of the cardiovascular and respiratory systems during exercise is altered (Crisafulli et al., 2009).

In the present study, we have evaluated the effectiveness of a pHPMA hydrogel as a implant material to bridge the defected spinal cord tissue and to facilitate axonal regeneration. A great attention was paid to neurophysiological changes that may be related to the development of enhanced excitability of hind limb reflexes after a SCI. Thus, functional recovery was assessed by means of two tools–Hoffman (H)-reflex and respiratory adjustments during muscle induced fatigue. In addition, qualitative morphology of the injury site was assessed by estimating the white matter myelin preservation, the number of macrophages/monocytes (ED-1 positive cells) at the lesion border and the neurofilament-positive (NH-F) axon profiles penetrating the hydrogel.

## MATERIALS AND METHODS

### Animals

Adult male albino Sprague Dawley rats (8 weeks old at the start of the experiment), weighing 300 g (Elevage JANVIER®, Centre d’Elevage Roger JANVIER), were singly housed in smooth-bottomed plastic cages at 22°C in a colony room maintained on a 12-h light/dark cycle, with dark onset at 7.00 PM. Food (Purina®, rat chow) and water were available *ad libitum*. On arrival, all animals were examined by a qualified veterinarian. In order to accustom the animals to the laboratory environment, an acclimation period of 3 weeks was allowed before the initiation of the experiment. All animals were weighted before each experimental step.

### Ethical approval

Anaesthesia and surgical procedures were performed according to the French law on animal care guidelines and the Animal Care Committees of University Aix-Marseille (Aix-Marseille *Université*) and CNRS (*Centre National de la Recherche Scientifique*) approved our protocols. Individual conducting researches were listed in the authorized personnel section of the animal research protocol or be added to a previously approved protocol. Furthermore, experiments were performed following the recommendations provided in the *Guide for Care and Use of Laboratory Animals* (US Department of Health and Human Services, National Institutes of Health) and in accordance with the European Community's council directive of 24 November 1986 (86/609/ EEC). No clinical sign of pain or unpleasant sensation (i.e. screech, prostration, hyperactivity, anorexia) and no paw-eating behaviour were observed throughout the study. At the end of the experiments, animals were sacrificed by an intra-arterial overdose (1 ml) of sodium pentobarbital solution (Nembutal®, Sanofi Santé Animale, 60 mg kg^−1^).

### Experimental groups

A total of 35 animals were assigned to the following treatment groups: (i) unlesioned (control, *n*=10), (ii) hemisected non-grafted (lesion, *n*=15) in which the lesion cavity was left empty, (iii) hemisected and immediately implanted with a block of pHPMA hydrogel into the spinal cavity (Lesion+Matrix, *n*=10).

### Hydrogel preparation

Water-soluble matrix was provided by Dr Eric Pinet (AquaGel Technologies Inc.). Briefly, the macroporous hydrogels were prepared from HPMA by heterophase separation using radical polymerization in a pore-forming solvent with a divinyl cross-linking agent. The synthesis of the pHPMAH_3_CCH(OH)CH_2_NHCOC(CH_3_)=CH_2_ hydrogel was previously described (Woerly et al., 1998). Following polymerization the gel was extensively washed in water to remove unreacted products and then stored in distilled water at 4°C. Prior to its use the hydrogel was sterilized by exposure to hot dry air (+121°C) in autoclave for 15 min.

### Hydrogel characteristics

pHPMA is a biocompatible synthetic hydrogel that displays no signs of toxicity, chronic inflammation or risks of transmitting disease (Woerly et al., 2001a). This hydrogel is a cross-linked hydrophilic macromolecular network that has a water fraction of 95.66%. Its mechanical properties, matching those of the nervous system, allow it to properly fit the lesion cavity and bridge the spinal cord stumps (Woerly et al., 1998). Moreover, this hydrogel presents a multimodal pore size distribution from micropores (<2 nm) to macropores (>50 nm; <300 μm) (Woerly et al., 1998). In addition, the interconnected pores allow free transport of macromolecules, migration of cells and angiogenesis (Woerly, 2000). It was also observed that axonal regeneration could cross throughout the hydrogel (Woerly et al., 2004). The large surface/volume ratio highly encourages tissue formation into the hydrogel (Woerly, 2000).

### Surgical protocol

For the initial surgery, rats were deeply anaesthetized with an intra-peritoneal injection of chloral hydrate (400 mg kg^−1^, Sigma) and additional (0.1 ml) anaesthesia was given approximately every 20 min. Atropine (0.2 ml, subcutaneous) was administered to reduce secretions. Surgical procedures were performed aseptically with the aid of dissecting microscope. Rats were then positioned in ventral decubitus. The animal's back was shaved, disinfected (betadine, 5%) and a midline incision was made over the C6-T13 spinous processes. Dorsal muscles were cut and maintained on the side using retractors while spinous processes of the vertebra T10 were partially removed. Thoracic spinal column was fixed with the help of haemostatic forceps. A hole was made in the T10 vertebra arch using a Friedman micro-rongeur. After bone removal, periosteal membrane was opened with a pair of sharp tweezers to allow direct visualization of the spinal cord. The dura was opened longitudinally along the midline and retracted laterally to expose the dorsal surface of the cord. Prior to the transection, the spinal cord was rinsed with cold saline to favour vasoconstriction. Using a microscalpel, a left hemicordotomy was performed and 1 mm of spinal tissue segment was removed with microforceps and ophthalmic sponges. Close examination of the cut edges of the cord confirmed that the surface of the tissue was free of meninges or blood clots. The hydrogel implant was sized and adapted to the dimension and shape of the hemi-cavity. It was introduced into the lesion with a slight downward pressure to allow settling of the gel using a microspatula and ophthalmic sponges ensuring apposition of the polymer gel with the rostral, caudal and sagittal surfaces of the spinal tissue. The gel was hydrated with drops of sterile saline solution to ensure complete apposition of the surfaces between the polymer implant and the cord stumps (which was verified under the surgical microscope under high magnification). The surgical site containing the polymer implant was covered with a film of bioabsorbable artificial dural substitute (Seamdura®, Gunze Ltd., Codman, Johnson and Johnson Company) to isolate the gel implant from the overlying muscle and mesenchymal tissue. Muscle and skin were sutured (Vicryl® 3-0, Ethicon France) in anatomical layers. Animals were kept under heat lamps for 12–24 h. They were rehydrated with a bolus of saline (3 ml, subcutaneous) to replace fluid lost during the surgical procedure and received a subcutaneous injection of an antalgic (buprénorphine, 0.05 mg kg^−1^). Then, they were kept in individual cages and observed daily during the next 14 weeks. The animals were not placed in an enriched environment. They were preventively treated (in their drinking water) with wide spectrum antibiotic (Oxytetracycline, 400 mg l^−1^, Sigma Aldrich, Saint-Quentin Fallavier) during 1 week. Manual bladder expression was performed at least twice daily until bladder reflex was re-established (10-14 days post-surgery).

### BBB test

The animals were observed for any spontaneous recovery of motor activity and postural function of their hindquarters during overground movement. Behavioural evaluation was performed in an open field, once a week for 14 weeks, according to the BBB scale (Basso et al., 1995). This locomotor rating scale designed to measure the amount of locomotor function evaluates the movements of the rats on a 21-point rating scale, with a score of 21 representing normal movement and 0 representing complete paralysis of the hindlimb. This test is based on the normal recovery observed after a mild SCI and involves detailed analysis of movement including the motion degree of particular joints of the hindlimbs, trunk stability during movement, weight-bearing capabilities and the placement of each paw of the hind limbs.

### Recording protocol

Fourteen weeks post-surgery, rats were re-anaesthetized by an intra-peritoneal injection of solution containing urethane (120 mg kg^−1^, Sigma). A tracheotomy was performed. A catheter was inserted into the right femoral artery and pushed up to the fork of the abdominal aorta. This catheter, which allowed one to inject additional anaesthesia (approximately 50 mg kg^−1^ every hour) to suppress voluntary movement and whisker tremors but maintain corneal reflex, was positioned in order to let the blood flow freely to the left lower limb muscles. Animals were placed on a stereotaxic apparatus (Model 902, David Kopf Instrument). The left peroneal nerve was dissected free from surrounding tissues on a length of 3-4 cm and tendons were left intact. Animals were then prepared for reflex testing. Urethane does not depress spinal cord monosynaptic reflexes and does not alter presynaptic inhibition. This anaesthetic has been shown to produce similar negligible effects on H-reflex as ketamine, which is commonly used for H-reflex recordings (Cliffer et al., 1998). Core temperature was maintained at 36±1°C using a heating pad controlled by a thermostat driven by a rectal probe. To prevent dehydration, subcutaneous injection of 0.5 ml of isotonic glucose–saline, pH balanced to 7.4, were administered at 0.5 h intervals. The animal's forelimbs and hindlimbs were secured with tape while knee and ankle joints were firmly held by clamps on a horizontal support to prevent any movement during nerve (H-reflex testing) or muscle (physiological reflexes testing) stimulation. Hindlimbs were only slightly extended away from the trunk, and care was taken not to apply any unnecessary pressure or stretch to any part of the limbs.

### M- and H-waves

A bipolar cuff electrode was placed on the peroneal nerve for stimulation (0.1 ms pulses). The cathode (positive electrode) was oriented proximally. A pair of wire electrodes was placed subcutaneously on the *Tibialis anterior* muscle for EMG (electromyographic) recording. Ground electrode was implanted in a nearby muscle. Exposed tissue was covered with paraffin oil to prevent drying. The recorded signal was passed to a differential amplifier (P2MP®, 5104B) and bandpass filtered at 0.1 Hz and 10 kHz. The analogue signal was then sent to an A/D converter and the digital waveform was stored and displayed (sampled at 20 KHz, filtered with High Pass at 150 Hz) online using data acquisition software (Biopac MP150® and AcqKnowledge® software). Stimulation of the peroneal nerve produced two EMG responses. The earlier response (< 4 ms latency), the M-wave, was due to direct activation of the motor axons in the peroneal nerve and did not involve a spinal circuit. The second response (> 4 ms latency), the monosynaptic H-reflex, was due to the activation of muscle afferents in the peroneal nerve that synapse on sciatic motoneurons. The H-reflex provides a quantitative measure of network changes that occur in the spinal cord after injury. More precisely, changes in the H-wave can be correlated to the amount of damaged grey matter (D’Angelo, 1973).

In order to elicit the H-reflex, the peroneal nerve was stimulated using a Grass® S88 stimulator (Astro-Med, Inc.) delivering bipolar electrical pulses of 0.1 ms duration. Threshold and maximal response amplitudes were measured. Stimulus intensity was gradually increased until both M- and H-waves were maximal and stable: in the low stimulus intensity, only M-wave was evoked, whereas the H-wave was observable at higher intensity. Thus, when the H_max_ was reached, the M-wave amplitude was already maximal. The total motor unit response was determined by supramaximal stimulation of the peroneal nerve axons to produce a maximal M-wave.

In a first step, the influence of supraspinal descending axons on the sub-lesional sensory-motor circuitry was examined. The H-reflex rate sensitivity (i.e. the decrease in reflex magnitude relative to repetition rate) was studied (Thompson et al., 1992; Skinner et al., 1996; Lee et al., 2005; Reese et al., 2006; Lee et al., 2009; Bianco et al., 2011). A control repetition rate of 0.3 Hz was used for H-reflexes consistent with the original studies describing rate-sensitive depression as a diminished response produced by successive stimuli falling at intervals of<3 s (Eccles and Rall, 1951). Although a small depression was subsequently shown to outlast this period, 3.0 s provided a practical compromise between recovery and efficient performance of the protocols (Lloyd and Wilson, 1957). The amplitudes of the M- and H-waves as determined by the peak-to-peak values of each waveform were used to calculate the average H_max_/M_max_ ratio. The latencies of the responses were measured as time elapsed between trigger and peak of each waveform. H_max_/M_max_ ratio provided an index of motoneurons recruited via a monosynaptic reflex relative to the total motoneuron pool (Magladery et al., 1951a, 1951b; Taborikova, 1966). Accordingly, the H-reflex magnitude, expressed as a fraction of the M-wave, provided a standard that could be referenced across animals. Thereafter, stimulation was performed at frequencies of 1, 5 and 10 Hz, with a 5-min inter-rate interval. The same stimulus intensity previously defined (i.e. the minimum stimulus intensity required to elicit maximal H-wave) was used across the applied frequencies. Immediately after changing to a new test frequency, the magnitude of the initial two or three reflexes progressively reached a new plateau. To decrease the variability that could be due to this transition, the signal average, at each test frequency, was recorded after the initial reflexes to exclude responses with the most acute transition. Fifteen consecutive waveforms were collected at each frequency and an average of ten responses was stored after discarding the first five responses in order to obtain an average of the stabilized reflex. The H_max_/M_max_ ratio at 1, 5 and 10 Hz was expressed as a percent of 0.3 Hz baseline frequency ratio in order to calculate the H-reflex rate depression. Following the frequency series testing, the H-reflex amplitude was confirmed at 0.3 Hz for consistency. If the amplitude at recheck was less than 90% of the initial amplitude, data were discarded. The M- and H-waves were only recorded on the left side. The investigator was blind to the experimental groups during recording and analysis of the data.

In a second step, the reflex excitability was tested using a modification of the method reported by Skinner et al. (1996). The H-reflex response to repetitive stimulation at 10 Hz was normally reduced by the second and subsequent stimuli, probably through presynaptic inhibitory mechanisms. However, in injured animals, the normal inhibition was absent, and the H-reflex amplitude remained close to 100% of its control value. Single square wave stimuli (0.5 ms, 5-15 V) was used to elicit M-wave and H-reflex and then trains of five stimuli at 10 Hz are delivered at 5x H-reflex threshold. As described by Gozariu et al. (1998) in rats, when increasing the stimulation intensity, the M- and H-waves reached a plateau so that at 5x the H-reflex threshold amplitude of M- and H-waves were maximal. The amplitude of the M-wave was monitored throughout the recording period to ensure it remained constant. H-reflex amplitude of the second, third, fourth and fifth responses were measured from the average of three trials and H_max_/M_max_ ratios (2nd, 3rd, 4th, 5th) were expressed as a percentage of the first H_max_/M_max_ ratio (1st), also averaged over three trials.

### Physiological reflexes

Changes in ventilation were recorded after ipsilateral *T. anterior* stimulation to the spinal lesion. The experiments were performed after regional circulatory occlusion, which isolated and maintained the neural drive as well as abolishing the humoral communication. In intact animals, repetitive muscle stimulations induced muscle fatigue responsible of the metabosensitive afferent fibre activation and subsequently a ventilation frequency and amplitude increase (Decherchi et al., 2007). In animal with an SCI, such stimulation was ineffective, i.e. the ventilation response was abolished. However, the changes in ventilation after muscle stimulation may reappear after an effective therapeutic treatment (Bianco et al., 2011).

One-hour of rest after the last nerve stimulation used to evoke M- and H-waves, EIF (electrically induced muscle fatigue) were performed. For this purpose, rhythmic muscle contractions were produced by a neurostimulator (Grass® S88, Quincy), which delivered, through an isolation unit, rectangular pulse trains to a pair of steel electrodes placed on the muscle surface (pulse duration: 0.1 ms; frequency: 10 Hz, i.e. five shocks in each 500 ms train; duty cycle: 500/1500 ms, voltage range: 5–8 V). The voltage was supramaximal, i.e. 20% higher than that used to elicit a maximal twitch. Fatigue was assessed from the decay of force throughout the 3-min EIF period. The strength of muscle contraction (the decay of force) was measured from the beginning to the end of this 3-min muscle electrical stimulation with a gauge strain attached to the distal tendon (Microdynamometer S 60; Ugo Basile Narco Biosystem). We chose to stimulate directly the muscle because it was previously shown that muscle low frequency stimulation is a strong activator of metabosensitive afferent fibres (Darques and Jammes, 1997). Ventilatory frequency recorded through the canula inserted into the trachea was measured 2 min before EIF, during EIF and 5 min after.

**Figure 1:**
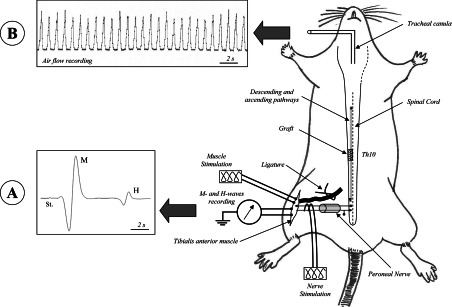
Recording protocol (**A**) Fourteen weeks post-surgery, the peroneal nerve was stimulated to elicit the M- and H-waves. The influence of supraspinal descending axons on the sub-lesional sensory-motor circuitry was examined and the reflex excitability was tested. (**B**) One-hour of rest after the last nerve stimulation used to evoke M- and H-waves, EIF were performed, under regional circulatory occlusion, in order to induce metabosensitive afferent fibre activation and subsequently a ventilation frequency and amplitude increase. Examples of M- and H-waves recording and air flow movements are given.

The recording protocol is summarized in Figure 1.

### Muscular weight

At the end of the electrophysiological recordings, animals were sacrificed by a lethal dose of sodium pentobarbital (Nembutal, intravenous 120 mg kg^−1^). The *T. anterior* muscles were harvested and immediately weighed on a precision scale (OHAUS, Navigator™, N30330 model). Comparisons of muscle atrophy were performed using a muscle weight/body weight ratio.

### Histology

A 10-mm long spinal cord segment centred at the injury site was quickly removed, fixed in 4% (v/v) paraformaldehyde in PBS (phosphate buffer saline) for 24 h and then rinsed for 3 times in PBS. Subsequently, for cryoprotection the fragment of cord was placed in 15% (w/v) sucrose in PBS for 3 h and in 30% (w/v) sucrose in PBS overnight. The sample was embedded in optimal cutting temperature compound, and frozen on dry ice. The spinal cord was serially sectioned by means of a cryostat (Carl Zeiss GmbH). Ten micrometer thick serial longitudinal sections were rehydrated in PBT [phosphate-buffered saline +0.2% (v/v) Triton X-100] for at least 20 min and then utilized in immunofluorescence studies.

### Immunofluorescence studies

For immunofluorescence studies, the sections were rinsed with PBS, treated with blocking solution and incubated with primary antibodies overnight at 4°C. Two primary antibodies were used: the mouse anti-rat macrophages/monocytes (MAB1435/CD68, 1:50, Chemicon-Millipore) to detect host lesion response (ED1) and the polyclonal chicken anti-rat NF-H (Neurofilament Heavy chain, AB5539, 1:600, Chemicon-Millipore) as a marker for axons. After treatment with primary antibodies, the sections were washed with PBS and incubated with secondary antibodies (Alexa Fluor® 555 goat anti-mouse 1:200 and Alexa Fluor® 488 goat anti-chicken 1:200, Molecular Probes®, Invitrogen, Life Technologies Italia) for 2 h at room temperature. Sections were washed in PBS, nuclei were stained with DAPI (4′,6-diamidino-2-phenylindole) (Hoechst 1/1000) and then mounted using the FluorSave Reagent (Calbiochem, Merck Chemical) and analysed by confocal microscopy. In control determinations, primary antibodies were omitted and replaced with equivalent concentrations of unrelated IgG of the same subclass.

### Assessment of myelin preservation

In order to perform a homogeneous analysis, fluoromyelin staining (FluoroMyelin Green, Molecular Probes, Invitrogen) was carried out on spinal cord sections from both Lesion and Lesion+Matrix groups placed on the same coverslip. Myelin preservation was evaluated comparing the levels of myelin in the ventral white matter at 0.4 mm (rostral and caudal) ipsilaterally from the lesion epicenter in both Lesion and Lesion+Matrix groups, since the preservation of descending and segmental motor pathways located in the lateral and ventral funiculi, which contains descending motor pathways, was shown to contribute substantially to locomotor function in rat which underwent SCI; we previously reported that the quantification of the spared ventral myelin evaluated in a semi-thin section gave comparable results when fluoromyelin was used (Vitellaro-Zuccarello et al., 2007). The choice of the ventral white matter was based on the knowledge that the reticular spinal pathway descends mostly in the ipsilateral dorso- and ventrolateral funiculi and is directly involved in the regulation of the movement of the animal foot (Vitellaro-Zuccarello et al., 2007). The confocal microscope (Leica TSC2; Leica Microsystems) images for the two groups were obtained using the same intensity, pinhole, wavelength and thickness of the acquisition. As reference, we used sections close to the ones analysed and not treated with fluoromyelin. Briefly, the procedure of the staining was carried out by incubating the cryosections with Fluoromyelin diluted 1:300 in PBS for 20 min; slides were then washed 3 times for 10 min each with PBS and mounted with FluorSave (Merck, Darmstadt), and qualitatively and quantitatively analysed.

### Estimate of the macrophages/monocytes number at the site of lesion

ED1-positive cells were counted in transversal sections in a region of 4 mm centred at the lesion site. As negative reference for the confocal analysis we used a consecutive section that was stained omitting the primary antibody anti-rat macrophages/monocytes and replacing it with equivalent concentrations of the unrelated IgG of the same subclass. The zero level was adjusted on this reference and used for all the further analysis (we used a new zero reference for each new staining). The ED-1 positive cells present in a group of three consecutive sections (10 μm thick) were averaged, and we repeated this count each 100 μm. The total number of ED1-positive cells was obtained integrating the average within the volume analysed, i.e. the 4 mm around the epicenter of the lesion (Coggeshall, 1992).

### Statistical analysis

Data processing was performed using a software program (Instat® 3.0, GraphPad software). Data were expressed as mean±S.E.M. and were compared by ANOVA (paired, two-tailed) or *t* tests. The groups were examined for linear trends and *post-hoc* group comparisons were performed with Student Newman Keuls multiple comparisons post-test. Results were considered statistically significant if the *P*-value fell below 0.05.

## RESULTS

### Behavioural study

**Figure 2:**
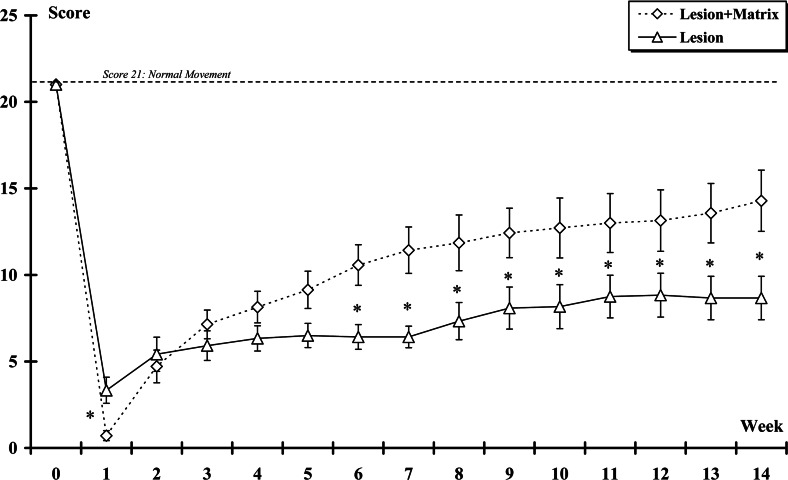
Locomotor evaluation according to the BBB scale The score was plot over time after spinal hemisection for all rats in each experimental group. Twenty-one represented normal locomotion, whereas zero represents no observable limb movement or weight support. From the 6 weeks some significant differences appeared between the grafted and non-grafted animals; the animals from the Lesion+Matrix group showing a higher recovery. (*: Lesion group *vs* Lesion+Matrix group. *, *P*<0.05).

In all animals, the severity of the spinal injuries was determined by the BBB locomotor rating scale that measured the amount of locomotor function. The average BBB scores of the rats at the first week were 3.3±0.75 and 0.71±0.29 in the Lesion and Lesion+Matrix groups, respectively. One week after the lesion, the score in the Lesion+Matrix group was statistically lower (*P*<0.05) than those of the Lesion group. Fourteen weeks after surgery, the BBB scores of the rats were 8.66±1.25 and 14.29±1.77 in the Lesion and Lesion+Matrix groups, respectively; the score of the implanted group being significantly higher (*P*<0.05) than the non-implanted one. Furthermore, the rats showed functional improvement after injury, which is typical of animals recovering from spinal shock (Gale et al., 1985), reaching a plateau for the Lesion group at the second week post-surgery (Week+2) but showing gradual improvement over 6 weeks for the Lesion+Matrix group. BBB scores evolution are presented in Figure 2.

### Changes H-reflex responses

Fourteen weeks post-surgery, the mean latency of the M-wave was 2.33±0.09 ms, 2.15±0.06 and 2.38±0.08 ms for the Control, Lesion and Lesion+Matrix groups, respectively. The mean latency measured in the Lesion group was significantly (*P*<0.05) lower than the mean latencies found in the two other experimental groups. The latency of the H-reflex was 5.35±0.14, 5.96±0.09 and 5.71±0.13 ms in the Control, Lesion and Lesion+Matrix groups, respectively. The mean latency measured in the Lesion group was significantly higher than the mean latencies found in the Control (*P*<0.01) and Lesion+Matrix (*P*<0.05) groups. Although higher than the Control group, the mean latency of the Lesion+Matrix group was found not significantly different.

H-reflex recorded at 0.3 Hz stimulation rate was used to calculate the baseline H_max_/M_max_ ratio. Animals from the Lesion group had a significant (*P*<0.05) lower baseline H_max_/M_max_ ratio (0.36±0.03) than Control (0.48±0.14) and Lesion+Matrix (0.48±0.06) groups. No difference was found between the Control and Lesion+Matrix groups.

**Figure 3:**
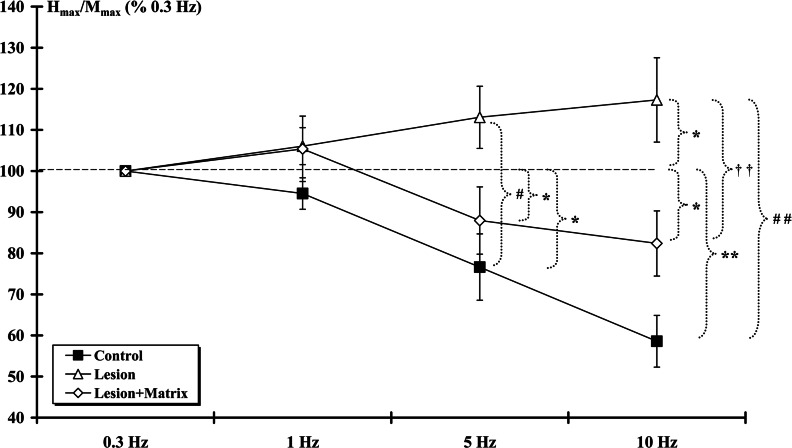
H-reflex rate sensitivity At the 14th week post-surgery, frequency-dependent values of H-reflexes were assessed. The H_max_/M_max_ ratio at 0.3 Hz frequency was assigned as 100% response. At 5 and 10 Hz stimulation, ratios of Control and Lesion+Matrix groups were statistically similar and the decrease was statistically significant, contrary to Lesion group. (*, Control and Lesion+Matrix groups against 100%; #, Control group against Lesion group; †, Lesion group against Lesion+Matrix group. *, *P*<0.05; **, *P*<0.01; #, *P*<0.05; ##, *P*<0.01; ††, *P*<0.01).

Given that H_max_/M_max_ ratio was constant across groups at 0.3 Hz frequency, the frequency-dependent value of H-reflexes was assessed (Figure 3). The H_max_/M_max_ ratio at 0.3 Hz frequency was assigned as 100% response. As the frequency of stimulation was increased to 1 Hz the ratio slightly decreased for the Control (94.53±3.82%) and sli-ghtly increased for both Lesion (106.06±4.49%) and Lesion+Matrix (105.40±7.96%) groups. These changes were not significantly different. Moreover, no statistical difference was found between groups. At 5 Hz stimulation, a significant (*P*<0.05) decrease was observed in both Control (76.63±8.06%) and Lesion+Matrix (87.96±8.16%) groups while no significant difference was shown in Lesion (113.07±7.56%) group. Comparison between groups indicated a significant difference (*P*<0.05) between the Lesion and the Control groups. At 10 Hz stimulation, the ratios of Control (58.56±6.29%) and Lesion+Matrix (82.38±7.92%) groups decreased significantly (*P*<0.01 and *P*<0.05, respectively). Although higher, the ratio measured in the Lesion+Matrix group was similar to those of the Control group. In the Lesion group the ratio (117.29±10.25%) increased significantly (*P*<0.05). The Control and Lesion+Matrix groups were significantly (*P*<0.01) lower from the Lesion group.

Therefore in the Control animals, the H_max_/M_max_ ratio steadily decreased with increasing stimulus frequency. However, after SCI, the rate depression property of the H-reflex was insensitive to stimulus frequency in the Lesion group, as indicated by the higher percent of Control and Lesion+Matrix groups H_max_/M_max_ ratio values.

**Figure 4:**
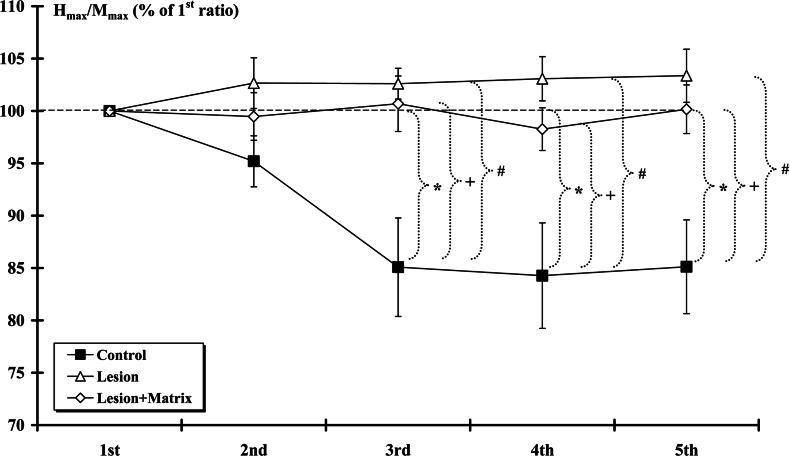
H-reflex excitability At 14th week post-surgery, H-reflex responses at 10 Hz-train stimulation were markedly reduced by the second and subsequent stimuli (normalized to 1st response) in Control groups. This depression was absent in rats from Lesion and Lesion+Matrix groups. (*: Control group against 100%, #: Control group against Lesion group, +: Control group against Lesion+Matrix group. *, *P*<0.05; #, *P*<0.05; +*P*<0.05).

Measures of the H-reflex responses to repetitive stimulation at 10 Hz are shown on Figure 4. Data indicated that H-reflex responses were markedly reduced (*P*<0.05) by the second and subsequent stimuli (normalized to 1^st^ response) in the Control group. This rate-sensitive depression was absent in rats from injured groups and there was no statistical difference between the Lesion and the Lesion+Matrix groups. Furthermore, a significant difference (*P*<0.05) was found between the Control group and the two others for the 3^rd^, 4^th^ and 5^th^ responses.

### Muscle force, weight and ventilatory response to fatigue

After 3-min stimulation (EIF) of the ipsilateral side of the spinal lesion, measurement of the decay of force throughout the 3-min EIF period indicated a force loss of −50.04±2.93% in the Control group against −69.05±3.14% and −65.17±4.12% in the Lesion and Lesion+Matrix groups, respectively. The Lesion and Lesion+Matrix groups presented the greater force loss (*P*<0.01) compared with the Control group. No significant difference was observed between the Lesion and the Lesion+Matrix groups.

The relative weight of the *T. anterior* muscle markedly decreased on the side of the SCI; the Lesion (1.42±0.05 g kg^−1^) and the Lesion+Matrix (1.41±0.05 g kg^−1^) groups showing a significantly (*P*<0.01) lower muscle weight than the Control (1.66±0.06 g kg^−1^) group. Between the operated groups, the relative weight of the *T. anterior* muscle was not different. There was also no difference between the relative weight of the muscle on the unlesioned side (Control: 1.68±0.06 g kg^−1^; Lesion: 1.65±0.06 g kg^−1^; Lesion+Matrix: 1.67±0.06 g kg^−1^).

**Figure 5:**
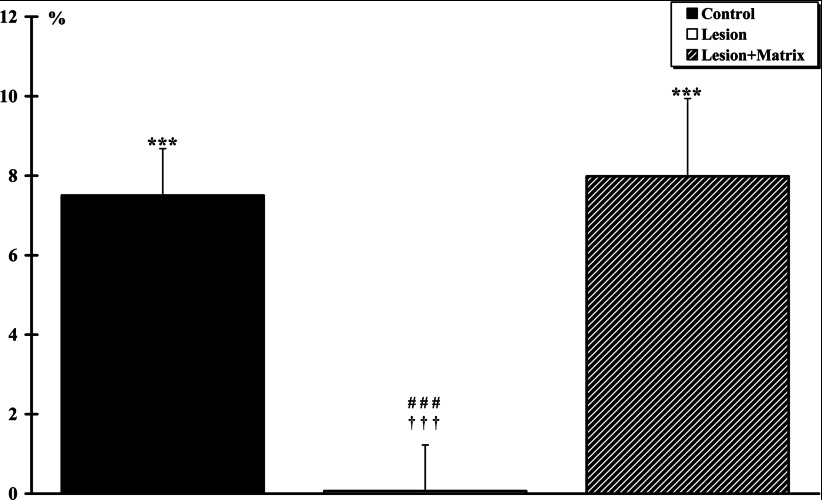
Ventilatory activity after a 3-min electromyostimulation The *T. anterior* muscle of the left side (ipsilateral side of the lesion for the Lesion and Lesion+Matrix groups) was stimulated. The ventilatory activity significantly (***, *P*<0.001) increased in the Control and Lesion+Matrix groups, respectively. No significant ventilatory increase was observed in the Lesion groups after muscle fatigue. Inter-group comparison reveals a highly significant difference between Lesion group and the two other groups (###, *P*<0.001 for the Control group and †††, *P*<0.05 for Lesion+Matrix group).

Air flow movements, recorded through the canula inserted into the trachea, represents the ventilatory activity for each animal (Figure 5). The ventilatory activity significantly increased by +7.50±1.18% (*P*<0.001) and +7.98±1.95% (*P*<0.001) in the Control and Lesion+Matrix groups, respectively. No significant ventilatory increase (+0.07±1.15%) was observed in the Lesion group after muscle fatigue. Inter-group comparison revealed no significant difference between Control and Lesion+Matrix groups. However, a highly significant difference (*P*<0.001) between Lesion and the two other groups was found.

### Morphological evaluation

**Figure 6:**
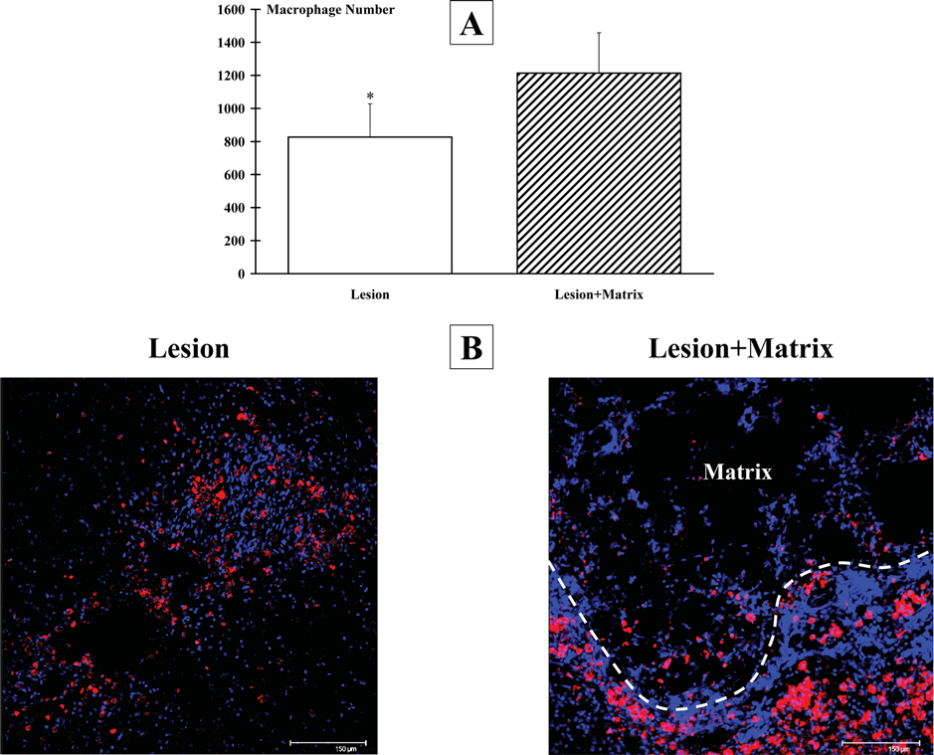
ED-1 positive cells Labelled cells were quantitatively estimated by means of ED1-antibody immunostaining and quantitative confocal microscopy. (**A**) Analysis of the host inflammatory response, performed 4 mm across the lesion site epicenter, showed that the infiltration of macrophages/monocytes was higher in the Lesion+Matrix group than in Lesion group. (**B**) ED-1 labelled cells (showed in red) accumulated mostly at the border of the lesion also when the matrix was applied. Cell nuclei are stained in blue with DAPI. Images shown are representative of the observed pictures. Scale bar: 150 μm (*, *P*<0.05).

**Figure 7:**
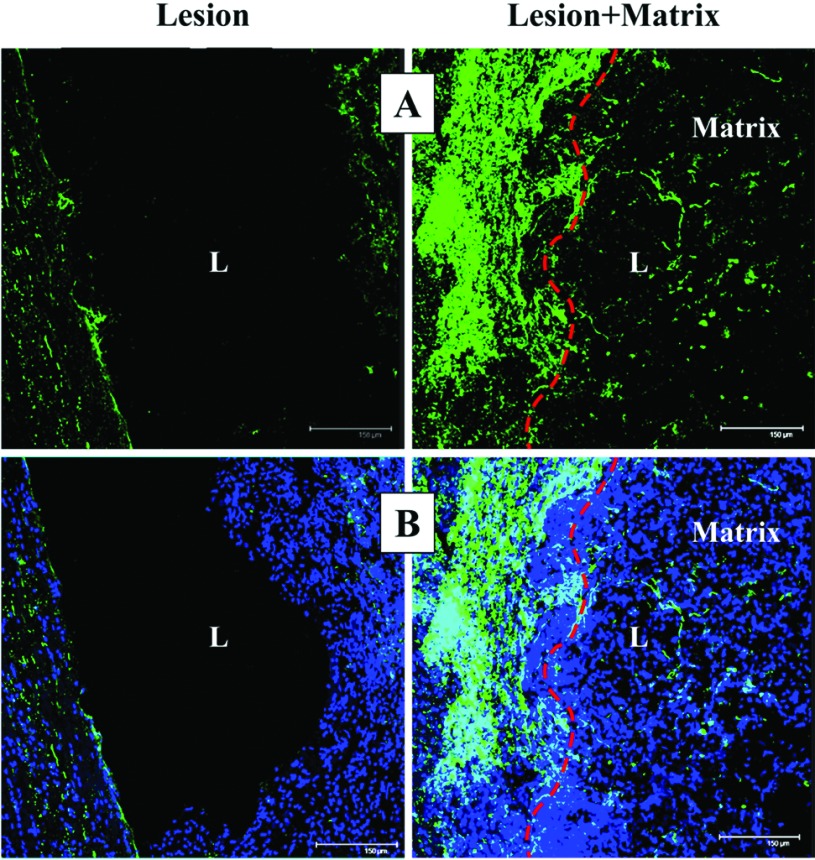
Axonal staining (**A**) Green NF-H-stained axonal profiles were accumulated at the lesion (L) edge, and deeply penetrate throughout the lesion when matrix was applied. (**B**) Combination of green NF-H-staining and blue DAPI-cell nuclei staining. Scale bar: 150 μm.

Analysis of the lesion cavity directly from the collected slices indicated that the lesion extent was comparable in both hemisected groups. Macrophages/monocytes infiltration at the site of injury was investigated by confocal quantitative estimation of the number of ED-1 immunostained cells in the examined 4000 μm. The number of ED-1 positive cells at the lesion site resulted significantly (*P*<0.05) lower in Lesion (827±201) than in Lesion+Matrix (1214±244) group. Some ED-1 positive cells penetrated into the matrix, but most of them were located in prevalence at the edge of the injury (Figure 6). A similar outcome was observed when neutrophil infiltration was investigated (results not shown). Numerous NF-H-positive axonal profiles were observed at the edge and throughout the matrix (Figure 7).

**Figure 8:**
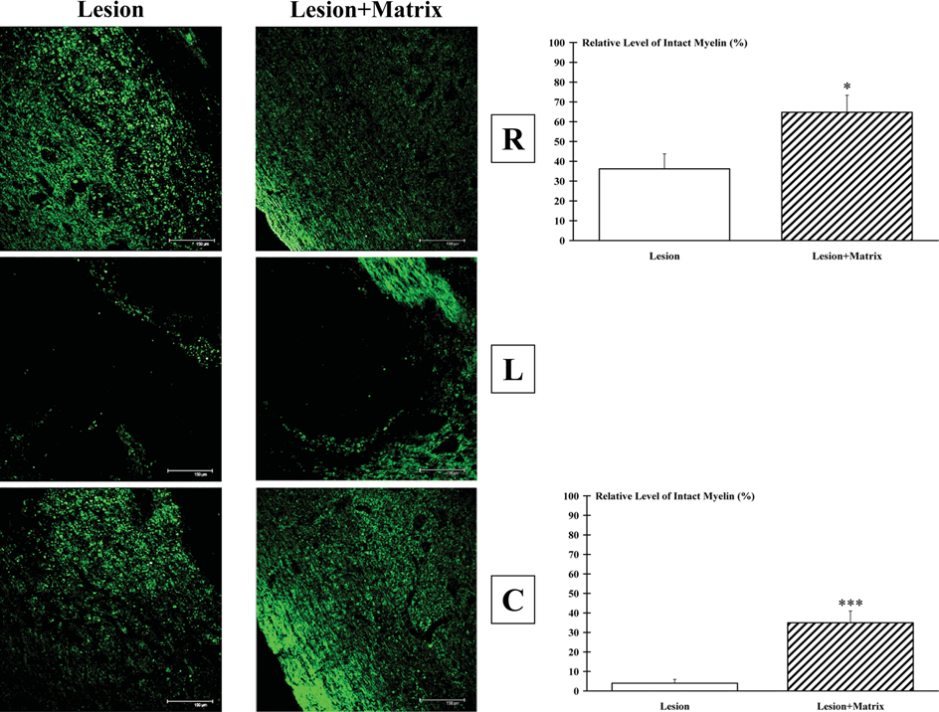
Myelin preservation Green FluoroMyelin staining showed globular bodies (degenerated myelin) both rostrally (R) and caudally (C) to the lesion (L) epicenter. The extent of such degeneration was much reduced when matrix was applied. The higher (*P*<0.05) sparing effect of matrix compared with injured spinal without matrix was particularly evident in rostral sections (R). Scale bar: 150 μm.

An important pathway in eliciting locomotion is the reticulospinal tract that descends mainly in the ipsilateral dorso- and ventrolateral funiculi. By electrophysiological studies, it was shown its function in the coordination of rhythmic stepping movements (Mori et al., 1998; Ballermann and Fouad, 2006). In this region, myelin preservation was studied by confocal analysis (details in the Materials and Methods section). FluoroMyelin stained cryosections indicated a much higher number of globular bodies (degenerated myelin) in both rostral and caudal cord samples of Lesion group compared with Lesion+Matrix animals (Figure 8). Quantification of retrogradely preserved myelin in rostral cord sections of the Lesion group resulted significantly lower (*P*<0.05) than in the Lesion+Matrix group. Moreover, the same analysis performed caudally to the lesion site showed that the protective effects of the matrix were significantly much higher, with a preservation stating to the 35±6% (*P*<0.001) (Figure 8).

## DISCUSSION

The present study shows, in an animal model of spinal cord hemisection, that a biocompatible macroporous pHPMA is a matrix that can (1) improve locomotor function based on BBB locomotor rating scale, (2) support axonal and myelin preservation, (3) improve respiratory response to electrically induced muscular fatigue and (4) restores H-reflex evoked response.

Compared with the Lesion alone group in which (1) the BBB score increases only during the first two weeks post-injury and reach a plateau, (2) the respiratory and the H-reflex response are not improved, there is no doubt that the hydrogel brings a suitable environment that allows a better functional recovery.

### Improved locomotor activity

We report in the current study that there was a significant improvement of the locomotion and the activity of the sensorimotor loops after reconstructive surgery of the lesion using pHPMA hydrogel implants. The functional loss due to the spinal cord hemisection was maintained throughout the entire time of the study in both groups. However, contrary to the Lesion group that reached a plateau at the second week post-surgery suggesting typical reorganization observed after an SCI (Gale et al., 1985), the Lesion+Matrix group presented a functional improvement until the sixth week. As in our study, previous experiments showed that after a spinal hemisection at the T9–T10 level a spontaneous locomotor recovery was observed (Teng et al., 2002). However, in a model of T8 dorsal hemisection that interrupts several pathways including the corticospinal and the raphespinal tracts, it was showed at 6 weeks after implantation of a biodegradable hydrogel (acrylated PLA-b-PEG-b-PLA) that the BBB score reached higher values (Piantino et al., 2006). Improvement of the functional motor recovery after pHPMA implantation was also observed following both a T8–T9 compression in rat (Woerly et al., 2001a) and a complete spinal cord transaction in cat (Woerly et al., 2001b; Woerly et al., 2004). In addition, motor improvements were also reported after implantation of pHPMA hydrogel containing RGD (Woerly et al., 2001c; Hejcl et al., 2010).

The functional outcomes after SCI were enhanced in the animals that were treated with a hydrogel suggesting the effectiveness of hydrogel technology.

### Improved response to fatigue

Here we show an abolishment of the adaptive ventilatory response after a hemitransection on the ipsilateral side and a recovery (i.e. increase in ventilation rate after muscle stimulation) in implanted rats. As we reported previously in a bilateral T10 spinal cord compression model (Bianco et al., 2011), the results obtained in the Lesion+Matrix group indicate a more effective recovery of the neural mechanisms involved in cardio-respiratory adjustments to muscular activity that can be initiated by the *T. anterior* stimulation (Decherchi et al., 2007). The afferent arm of this reflex is composed of thinly myelinated group III and unmyelinated group IV sensitive fibres that inform, throughout the spinal pathway, the medullary respiratory centres of the muscle contraction rate. More precisely, these sensitive fibres make their first synapse on spinoreticular tracts neurons in the dorsal horn of the spinal cord (Mitchell et al., 1977; Mitchell et al., 1983; Iwamoto et al., 1984; Iwamoto et al., 1985; Kozelka and Wurster, 1985; Bauer et al., 1989). Part of the ascending projections arising from the dorsal horn cells excites neurons in the cardiovascular control centre of the ventrolateral medulla that in return excite sympathetic preganglionic neurons located in the intermediolateral area of the spinal cord (Wilson et al., 1996) and probably, as we previously suggested, excites other subnucleus of the NTS (nucleus tractus solitarii) projecting on respiratory nuclei that could be seen as premotor respiratory neurons (Decherchi et al., 2007).

Our present results strongly suggest that when pHPMA hydrogel is implanted in lesioned spinal cord cavity, there might be a sensori-motor reorganization that allows the transmission of the sensory messages from the sub- to the supra-lesional spinal cord. This improvement may have an anatomical basis, i.e.: axonal regrowth throughout the lesion, myelination of the newly formed axons, synapse development (newly formed contacts between subpopulations of neurons) and/or reorganization of segmental, intersegmental and suprasegmental axonal circuitries. Previous studies showed that pHPMA hydrogel enabled the integration of the CNS cellular component into the lesion and promoted tissue restoration of the spinal cord (Woerly et al., 1999, 2001a). This reparative neo-histogenic process was made possible because the polymer gel established a stable structural three-dimensional continuity across the lesion cavity that served as a growth template that facilitated tissue morphogenesis, organization (infiltration of host cells within the pore structure of the gel, formation of tissue trabeculae) and axonal growth within the polymer implant (these axons were myelinated by ectopic Schwann cell that may have migrated into the lesion/implantation site). Thus, it had been suggested that pHPMA hydrogel had a strong stimulating effect on neurons and Schwann cells activity as well as supporting revascularization of the lesion site and having an apparent protective effect against Wallerian degeneration (Woerly et al., 1999, 2001a, 2001b). Finally, it had been suggested that because of its physico-chemical properties, the pHPMA hydrogel may have therapeutic interest in the repair of spinal cord lesion (Woerly et al., 1990, 1996a, 1996b) compared with various materials used to experimentally bridge the spinal cord lesion such as collagen matrix (Marchand and Woerly, 1990), guidance channels (tubes or cylinders) of poly(acrylonitrile-vinylchloride) (Xu et al., 1995), polycarbonate (Montgomery et al., 1996), poly(α-hydroxyacids) (Gautier et al., 1998; Oudega et al., 2001), silicone (Borgens, 1999), polyethylene glycol (Shi et al., 1999), poly(lactic acid) (Hurtado et al., 2011) and poly(lactic-*co*-glycolic acid) (Moore et al., 2006; Chen et al., 2009).

### H-reflex responses

Our study indicates that at the 14th week after hemisection of the spinal cord, the stimulation rate-sensitive H-reflex depression was absent in the Lesion group contrary to the Control and Lesion+Matrix groups in which the H-reflex responses were markedly reduced by the second and subsequent stimuli (normalized to 1st response). Our results also indicated that the rate sensitivity of the H-wave in the Lesion+Matrix group was close to control values. However, the H-reflex depression was absent at 10 Hz-train stimulation in the Lesion and Lesion+Matrix groups.

We interpret these changes as consistent with the view that implanted pHPMA induced a beneficial network reorganization that allows the transmission of the motor messages from the supra- to the sub-lesional spinal cord and that SCI resulted in a marked alteration in the distribution of background excitation and inhibition, producing changes in motoneuron excitability.

It was previously described that SCI in mammals are followed by a ‘spinal shock’ period with muscle paralysis, flaccid muscle tone, and loss of tendon reflexes below the level of injury. Several weeks after injury, a spastic syndrome develops showing exaggerated tendon jerks, increased muscle tone, and muscle spasm (Hiersemenzel et al., 2000). This second step has been explained as a result of cortical and subcortical release of spinal cord reflex circuits connecting muscle and skin sensory afferents with intact interneurons and motoneurons (Engberg et al., 1968). Corticospinal projections from primary, premotor and supplementary motor areas have been implicated in the modulation of spinal reflexes by mechanisms of presynaptic inhibition onto afferent sensory projections and their synapses with interneurons and motoneurons (Petersen et al., 1998). The cortically driven regulatory activity ceases when descending pathways are interrupted by the injury, thus giving rise to enhanced activity of spinal segmental reflexes under the injury level. The SCI-induced changes in the H-reflex by (i) increasing the H-wave amplitude and H/M ratio at the baseline stimulation and (ii) reducing rate depression upon low-frequency stimulation. Diverse mechanisms may be involved in the post-traumatic H-reflex enhancement such as increase in motoneuron excitability due to reappearance of large persistent inward ionic currents, decrease of presynaptic inhibition of Ia afferents, enhanced post-activation depression of transmitter release in Ia terminals and reduced reciprocal inhibition by Ia terminals from antagonist muscles (Hultborn, 2003; Li and Bennett, 2003; Li et al., 2004; Frigon and Rossignol, 2006). It is commonly described that short-term alteration in reflex excitability, occurring within hours and days after SCI, can be explained by loss of supraspinal and propriospinal inputs, by alterations in receptors and channels in motoneurons and by presynaptic terminals modulating neuronal excitability and transmitter release (Little et al., 1999; Hultborn, 2003; Frigon and Rossignol, 2006). In contrast, long-term changes, requiring weeks or even months, are generally associated with structural synaptic plasticity, i.e. axonal sprouting and formation of new synaptic contacts between primary afferents or spared descending axons, on the one hand, and interneurons and motoneurons in the lumbar spinal cord, on the other hand.

An unexpected observation in rats has previously been reported (Lee et al., 2005): rats with mild contusion injury, and thus fairly good functional recovery, display a decrease in the rate depression of the H-reflex, i.e. becomes more abnormal between the 1st and the 8th post-operative weeks. In contrast, the same authors reported that the depression increases, i.e. becomes more ‘normal’, in rats with complete transection of the spinal cord, an injury leading to poor functional outcome. The authors concluded that the H-reflex rate depression depends of the type and severity of the SCI (contusion against complete transection). They suggested that the H-reflex recovers some sensitivity to afferent stimulus frequency in the absence of any supraspinal connections. However, when supraspinal connections are partially spared, there is a further loss in sensitivity of the H-reflex to afferent stimulation frequency. After a complete spinal cord transection an increase in GAD (glutamic acid decarboxylase) and GABA (γ-amino-butyric acid) is observed (Smith et al., 1976; Tillakaratne et al., 2000; Tillakaratne et al., 2002). Since presynaptic inhibition of Ia afferents is GABA-mediated, increased GABA could explain the higher depression rate observed over time after transection. More recently Rudomin and Schmidt (1999), Lee et al. (2009) also showed that better functional recovery in mice is associated with enhanced H-reflex responses, an unexpected finding regarding the general view that hyperexcitability is a negative pathophysiological factor. These authors thus suggested that reduction in the rate sensitivity of the H-reflex provides functional advantages after incomplete SCI (compression) in mice.

The Lesion group presents the lowest baseline H_max_/M_max_ ratio 14 weeks after the hemisection. This result was related to a decrease in H-wave amplitude whereas the M-wave amplitude was maintained in any of our experimental groups. The presence of a decrease in the baseline H_max_/M_max_ ratio 14 weeks after the SCI seems to contradict previous reports. If baseline H_max_/M_max_ ratio was solely influenced by tonic inhibition from supraspinal pathways, then we should have observed an elevated baseline ratio if these descending pathways were interrupted. One previous study has reported an increase in the H-wave amplitude during the first couple of days after partial thoracic transection (80–95%) and a return to pre-injury values by the 3-4 days after injury (Chen et al., 2001). The authors suggested that plasticity induced by the presence of spared supraspinal or intraspinal pathways may explain these results after SCI. These results have also been attributed to changes in motoneuron number and/or morphology after SCI (Friedman et al., 1981; Zengel et al., 1983).

Although the deleterious consequences of an SCI can be observed many months after the trauma, we only analysed the short-term (14 weeks) effect of spinal cord hemisection at a thoracic level. The abnormal low baseline H_max_/M_max_ ratio observed in our Lesion group may reflect a motoneuron loss and/or changes in excitability properties in a context of severe SCI in which few descending axons were poorly or not functional. In the Lesion+Matrix group, the higher ratio (similar to the Control group ratio) may be explained by the beneficial effect of the implanted hydrogel, which may limit the glial scar formation, reform the tissue structure and help promote axonal regeneration across or around the lesion site. In the context of neuroplasticity alone, a decrease in secondary damage due to a less severe inflammatory reaction in the Lesion+Matrix group may also explain the observed functional improvement.

Several situations have been described in the literature. For example, a longitudinal study performed in tetraplegic patients reported an elevated *T. anterior* H_max_/M_max_ ratio associated with a decrease in M-wave amplitude (Hiersemenzel et al., 2000) while it was reported an increased ratio until 1 month after complete thoracic transection in the rat (Valero-Cabre et al., 2004). However, contrary to our study and others (Lee et al., 2005) in which only the peroneal branch was stimulated, they obtained their electrophysiological responses by stimulating the entire sciatic nerve which undoubtedly stimulated many muscles of the hindlimb that induced reciprocal facilitations between different muscle groups, as it was reported in SCI patients (Crone et al., 2003; Xia and Rymer, 2005). Some evidence indicated a correlation between 5-HT-IR and baseline H_max_/M_max_ ratio after incomplete SCI suggesting that spared descending serotonergic fibres may be involved (e.g. sprouting) in the recruitment of more motoneurons (Holmes et al., 2005; Lee et al., 2005). In our model, we can hypothesize that there is virtually no local serotonin as previously described after complete spinal cord transection (Schmidt and Jordan, 2000) and that the recovery of the H_max_/M_max_ ratio observed in the Lesion+Matrix group was mainly due to plasticity of spared descending pathways, or a regrowth of injured axons through or around the hydrogel, or changes in the motoneurons properties and 5-HT receptors.

### Axonal and myelin preservation

In this study, we confirm that the application of this type of matrix promotes axonal regrowth mainly supported at the edge of the matrix. Here we observed a higher presence of macrophages/monocytes (ED-1-positives cells), that may suggest a correlation between such axonal sprouting and the higher number of macrophages/monocytes. The higher number of ED-1-positive cells might be due to some retention action by the matrix. It is well known that the number of macrophages/monocytes has a peak around a week after spinal cord lesioning, and that later it decreases (Bottai et al., 2010). It is possible that the higher number observed in the Lesion+Matrix group might be due to a slower decrease compared with Lesion group rather than to an attractive action by the matrix itself. In addition, we show that matrix greatly counteracts the degeneration of myelin both rostrally and caudally to the lesion. The improved myelin preservation suggests a neuroprotective action by the matrix that seems to reduce the extent of secondary degeneration induced by the lesion. Such preservation occurs in the ventral white matter, where the reticular spinal pathway descends. This pathway regulates the movement of the rodent foot. Previous studies showed that pHPMA hydrogel implanted into the transected or compressed spinal cord cavity promoted the formation of a replacement tissue with histotypic characteristics of the host (Woerly et al., 2001a, 2001b). Indeed, the hydrogel that formed a stable bridge between the cord stumps was infiltrated by a reparative tissue composed of glial cells, capillary vessels, newly formed neurons presumably from precursor cells of the ependyma and/or migrating neurons and regenerating afferent and efferent axons (Woerly et al., 1998, 1999, 2001a, 2001b, 2001c).

### Conclusion

This work demonstrates that acute pHPMA hydrogel implantation into the hemisected T10 rat spinal cord induced locomotor and neurophysiological improvements. This recovery might be due either to a more suitable environment for regenerating axons and/or to a prevention of secondary injury and glial scar formation inducing higher neuroplasticity. The sub-lesional sensori-motor loops of grafted animals seem to be partially once again regulated by supra-lesional influences even though H-reflex excitability changes are still present 14 weeks after the SCI. Further studies are needed to determine which mechanisms are involved in the spinal cord plasticity after pHPMA hydrogel implantation. This study provides new insight into the concept that biomaterial technology constitutes a promising approach to repair spinal cord injuries.
